# Modeling Cryotherapy Ice Ball Dimensions and Isotherms in a Novel Gel-based Model to Determine Optimal Cryo-needle Configurations and Settings for Potential Use in Clinical Practice

**DOI:** 10.1016/j.urology.2016.02.012

**Published:** 2016-05

**Authors:** Taimur T. Shah, Uri Arbel, Sonja Foss, Andrew Zachman, Simon Rodney, Hashim U. Ahmed, Manit Arya

**Affiliations:** a Division of Surgery and Interventional Science, University College London, London, UK; b Department of Urology, University College London Hospitals NHS Foundation Trust, London, UK; c Whittington Hospital, London, UK; d Galil Medical Ltd., Yokneam, Israel; e Galil Medical Inc., Arden Hills, MN; f Princess Alexandra Hospital NHS Trust, Harlow, UK

## Abstract

**Objective:**

To gain a better understanding of ice ball dimensions and temperature isotherms relevant for cell kill when using combinations of cryo-needles we set out to answer 4 questions: (1) what type of cryo-needle? (2) how many needles? (3) best spatial configuration? and (4) correct duty cycle percentage?

**Methods:**

We conducted laboratory experiments to monitor ice ball dimensions and create multi-needle planar isotherm maps for 17G and 10G cryo-needles using a novel multi-needle thermocouple fixture within gel at body temperature. We tested configurations of 1-4 cryo-needles at duty cycles of 20%-100% with 1-2.5 cm spacing.

**Results:**

Analysis of various combinations shows that a central core of ≤−40°C develops at a distance of ~1 cm around the cryo-needles. Temperature increases linearly from this point to the ice ball leading edge (0°C), which is a further ≈1 cm away. Thus, the −40°C isotherm is approximately 1 cm inside the leading edge of the ice ball.

The optimum distance between cryo-needles was 1.5-2 cm, at duty cycle settings of 70%-100%. At distances further apart or with lower duty cycle settings, ice balls either had a central core >−40°C or had an hourglass shape.

**Conclusion:**

In answer to questions 1-3, tumor length, diameter, and shape will ultimately determine the number of needles and their configuration. However, we propose a conservative distance for cryo-needle placement between 1 and 1.5 cm should be adopted for clinical practice. In answer to question 4, using low duty cycle settings runs the risk of incomplete −40°C isotherm coverage of the tumor, and thus in routine practice we suggest that settings of 70%-100% are most appropriate.

Although cryotherapy has been in use for more than 50 years, recent advances have allowed it to develop into a potential treatment for both prostate and renal cancers.^1^, ^2^ Renal cryotherapy is already conducted in a focal tissue-preserving manner, and increasingly, cryotherapy is also applied for focal therapy of prostate cancer.^3^, ^4^ However, it is still an evolving technique that is adopted and effective in selected cases with local recurrence rates being higher in comparison with conventional radical surgery particularly in higher risk cases. A recent systematic review of focal prostate cryotherapy confirms good short- to medium-term oncological outcomes with a biochemical disease-free survival of between 71% and 93% and a minimal side effect profile for both primary and salvage cases.^5^ Renal cryotherapy is generally reserved for patients with small renal masses who are not suitable or unwilling to undergo partial or radical nephrectomy, and can be performed by either a percutaneous or laparoscopic approach.^6^, ^7^ Oncological outcomes are generally poorer with cryotherapy compared with partial nephrectomy, with a systematic review finding an increased relative risk of 9.39 for local progression. However, this was balanced against a significantly better side effect profile using cryotherapy.^8^

Earlier preclinical studies used older systems with liquid nitrogen as the cooling gas, whereas newer generation systems use compressed argon for cooling along with helium for thawing. Although there is extensive literature on the clinical outcomes of cryotherapy, largely from single-arm or multicenter registry studies, there is significantly less contemporary data evaluating the dynamics of ice ball formation using modern equipment. Thus, a potential reason for local recurrence may be the lack of information regarding ice ball isotherms (temperature gradient maps) particularly using multi-needle configurations, resulting in imperfect and incomplete cancer tissue ablation.

The developing ice ball normally has a leading edge of 0°C with a colder inner core. This temperature gradient map across the ice ball is made up of isotherms. Both liquid nitrogen and argon gas systems are theoretically able to produce temperatures close to −190°C. However, it is generally accepted that such low temperatures are not needed for cell death.

Various studies have assessed a so-called “lethal temperature” where complete cell death via coagulative necrosis is expected to occur. The currently accepted temperature is −40°C, beyond which further crystallization of water does not occur.^9^, ^10^

To ensure adequate ice ball coverage of the target volume at a sufficiently low temperature, a surgeon must understand 4 critical variables for the formation of an ice ball, namely (1) cryo-needle type, (2) number of cryo-needles, (3) cryo-needle configuration, and (4) duty cycle percentage (percentage on-off time).

The aim of our study was to investigate the interaction of these multiple variables in forming single and multiple isotherm maps, with the aim of improving pre- and intra-operative planning using modern cryo-needles and delivery systems. To do this, we developed a novel method for accurate testing of ice ball dimensions and isotherms. Subsequent use of these data in developing and improving cryotherapy technique may lead to better cancer cell kill and local control, and subsequently improved oncological outcomes.

## Methods

### Multi-needle Thermocouple (TC) Matrix Structure

**Figure 1:**
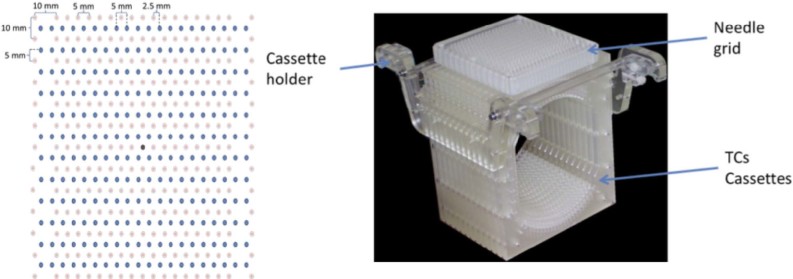
Assembled multi-needle thermocouple (TC) matrix. TC junctions (pink dots) and needle slots (blue dots). (Color version available online.)

A multi-needle TC matrix fixture was constructed from plastic frames and fine-gauge thermocouple wires to enable the recording of multiple-needle configuration isotherms. The exact configuration of the matrix is given in Figure 1. The placement holes allow for a two-dimensional configuration. The needles can be positioned as desired in the X-Y plane, which is parallel to the plane of temperature measurement (the needles are orthogonal to the measurement plane). The Z-axis (insertion depth) position is fixed to align the center of the ice ball with the measurement plane. This allowed us to place cryo-needles (each individually controlled) in any three-dimensional configuration using placement holes that were spaced every 5 mm in the X axis and 10 mm in the Y axis (similar to a template perineal grid used during cryotherapy) across the matrix.

### Cryo-needles

Three types of cryo-needles were evaluated: the IceSphere and the IceRod were 1.5 mm (17G) needles often used for prostate ablation, whereas the IceEdge was a larger 2.4 mm (10G) needle often used in larger organs such as for renal ablation (IceEdge). The IceSphere differed from the IceRod by having a shorter convective exchange region and thus would produce an ice ball 1.5 cm shorter in length.

Duty cycle describes the percentage of time cryogen gas is flowing through the needle. We tested 4 duty cycle settings of 100%, 70%, 50%, and 20%. In addition, needle configurations with 2, 3, and 4 needles placed at different spacing was evaluated (1 cm, 1.5 cm, 2.0 cm, and 2.5 cm).

Previous data reported the length of isotherms measured in gel at 21°C after a standard freezing procedure.^11^ However, because the body temperature is approximately 37°C, our study was performed in ultrasound (US) gel at 37°C to mimic the body heat load effect.

The single cryo-needle maximum ice ball diameter at 37°C was 3.4-3.6 cm for the 17G needles and 4.3 cm for the 10G needle. A smaller inner core of ≤−40°C had diameters of 1.8 cm (17G) and 2.4 cm (10G).

### Operating Procedure

The tested cryo-needles were placed in US gel at 37°C, with uniform temperature distribution maintained in the gel tank. All needles underwent a simultaneous standard freezing procedure of two 10-minute active argon freeze cycles separated by a 5-minute passive thaw. During the test, the planar temperature distribution at the largest ice plane (middle of the single ice ball height) was recorded by the multi-needle TC matrix fixture.

The recorded temperature distribution was analyzed to find and calculate the 0°C, −20°C, and −40°C isotherms at the center of the ice ball. The calculation was made using multi-needle analyzer software.

## Results

**Figure 2:**
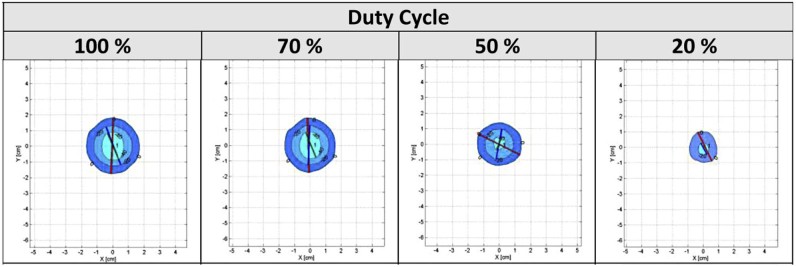
17G (IceRod), single cryo-needle dimensions with respect to duty cycle settings. (Color version available online.)

Initial single-needle tests assessing duty cycle percentage showed that when duty cycle was decreased to 70%, up to a 10% reduction was seen in the maximum ice ball diameter with an associated 10%-20% decrease in total volume and area of the −40°C isotherm. This effect was observed particularly with the 17G cryo-needles. As we dropped the duty cycle setting further to 50%, an associated 25%-50% decrease in maximum diameter and a 55%-75% decrease in volume of the −40°C isotherm were seen. At a setting of 20%, none of the cryo-needles were able to produce a −40°C isotherm (Fig. 2).

Subsequent testing of 2 cryo-needle configurations created either a cylindrical or an oblong ice ball with a maximum −40°C isotherm diameter of 3.1 cm (IceSphere), 4.2 cm (IceRod), and 4.7 cm (IceEdge). The 3 cryo-needle configurations created cylindrical ice balls until a distance of 2 cm where a pyramidal shape developed. The maximum lethal −40°C isotherm diameters were 4.4 cm for the two 17G needles and 4.9 cm for the 10G needle. Four cryo-needle configurations created larger cylindrical ice balls whose central core only rose above a temperature of −40°C at a distance of 2 cm apart, with the lowest duty cycle setting of 20%. The maximum lethal isotherm diameters were 4.7-5.0 cm for the 17G needles and 6.6 cm for the 10G needle.

**Figure 3:**
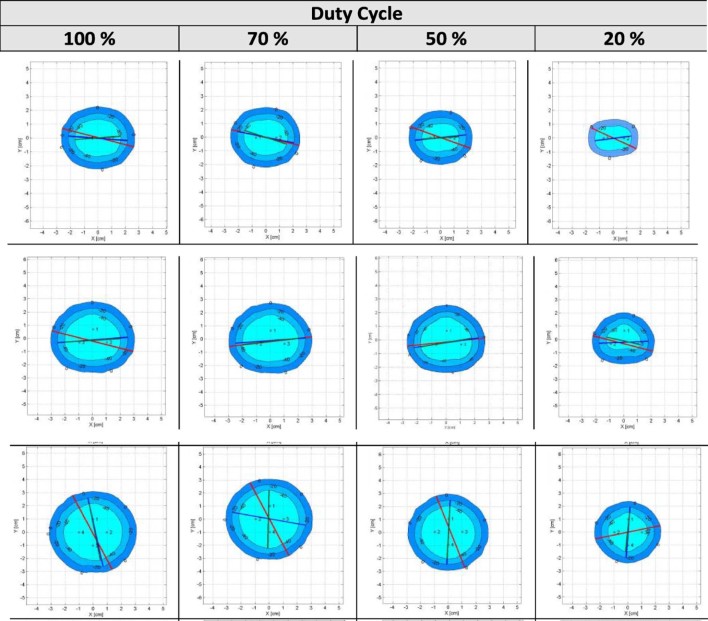
17G (IceRod), 2-needle (top), 3-needle (middle), and 4-needle (bottom) configurations at a spacing distance of 1.5 cm and varying duty cycles. (Color version available online.)

At a duty cycle setting of 100%, all cryo-needle configurations were able to create a confluent central core at distances of up to 2 cm apart. Only at the lowest duty cycle setting of 20%, as distance between the needles increased, an hourglass shape (loss of ice ball confluence) developed with the central core being warmer than the area closest to the cryo-needle. The most efficient distance for cryo-needle placement was 1.5 cm for the 17G needles and 2 cm for the 10G needle. The multi-needle maps with a spacing of 1.5 cm apart with varying duty cycle settings for the 17G IceRod needle are shown in Figure 3.

Analysis of the various combinations shows that a central core of ≤−40°C develops very consistently at a minimum distance of ~1 cm around the perimeter of the cryo-needles. The temperature increases linearly from this point to the ice ball leading edge (0°C), which is a further 0.85-1.15 cm away. Thus, the −40°C isotherm is approximately 1 cm inside the leading edge of the ice ball.

Analyzing duty cycle data shows us that, if needed, low duty cycle settings can be used to create small ice balls, but in routine practice the optimal is at a duty cycle of 70%-100%. This maximizes the area of the ice ball and allows consistent core temperatures of ≤−40°C at most configurations and distances.

## Discussion

Although numerous groups have tried gel, ex vivo, and in vivo modeling of the ice ball, to our knowledge, this is the first study to investigate so extensively the various ice ball dimensions and isotherms. This is also the first study to assess variations in duty cycle settings.

Our results show in all configurations the lethal −40°C isotherm was formed at a distance of 1 cm from the cryo-needle(s), and the leading edge of the ice ball was approximately a further 1 cm away. Single-needle performance deteriorates significantly at duty cycle settings of ≤50%, whereas multi-needle configurations have a synergistic effect with maximum efficiency noted at distances of 1.5 cm apart for the 17G needles and 2 cm apart for the 10G needle.

A major consideration when testing ice ball isotherms revolves around the thermal properties of live tissue versus water, gel, or ex vivo tissue. Many studies have used liver tissue, and although liver tissue has different thermal characteristics from water, it has been shown that ice balls created within 37°C water baths are relatively similar in size to those within the perfused liver.^12^, ^13^ Also, porcine liver has been shown to be relatively similar to cancerous tissue.^14^ Additionally, because of the higher water content of human tissue, thermal conductivity should not cause a major problem,^9^ although this may be different when ablation occurs near large blood vessels or air (lung) because of a heat sink effect.^15^, ^16^

Based on this information, we tested in a gel bath maintained at 37°C, which would be similar to but unlikely to precisely mimic in vivo human prostate or renal tissue. However, this model did allow us to test numerous combinations with relative ease while trying to maintain in vivo applicability. Performing 156 individual experiments would have been unfeasible and impractical using porcine models, and thus we felt that a gel-based model was the best compromise with the results and principles of ice ball isotherm formation still relevant for use in clinical practice.

Reviewing our results shows that they closely resemble a smaller study by Weld et al in live porcine kidneys.^17^ The authors also noted a synergistic effect with multiple cryo-needles, and to ensure a 1-cm margin, more than 1 needle needed to be used. Our data using the 17G IceRod 2 cm apart show that the lethal isotherm defined at −40°C was 1.8 cm using 1 needle, 4.4 cm using a 3-needle combination, and 5 cm using a 4-needle combination—this closely resembles their histologic ablative dimensions. We also found that the optimum distance between needles to maximize the ice ball area should be 1.5-2 cm, and a study in live porcine renal tissue has shown a similar result.^18^

A previous work by Saliken et al noted that the central core takes longer to fuse and may have lower temperature, which needs to be accounted for during clinical practice.^19^ Our results confirm this finding that when cryo-needles are further apart, a central core forms, which may not be at or below the lethal temperature of −40°C. Another factor to be considered is the uniformity of the ice ball. Although we did not notice this phenomenon in our experiments, others have found that the lethal temperature is not always uniform around the needles, and thus, it seems that for accurate in vivo lethal isoform measurements, multiple thermocouples should be routinely used.^20^ The distance of the leading edge from the lethal isotherm is also important and similar to a work by Littrup et al; in agar gel phantoms, they found that the lethal isotherm is smaller than total ice ball formed.^21^

Thus, when applying our results and also data from the reviewed literature into clinical practice, there are 4 factors that a surgeon needs to consider when performing cryotherapy, particularly with regard to the treatment field and ice ball configuration. The first is the type of cryo-needle used, and with regard to the prostate, the craniocaudal (base-apex) length of the prostate will ultimately determine the type of needle used. The length of the ice ball should not extend beyond the apex as this may increase the risk of injury to the urethral sphincter. With regard to the kidney, the maximum length of the tumor will determine which needle should be used.

As our results have shown, either focal or, if needed, large ice balls can be created by varying the remaining three factors: number of cryo-needles, needle configuration, and duty cycle percentage. Using this data set, a surgeon can accurately determine preoperatively which combination of factors he or she needs to cover his or her target lesion.

Similar to the ice ball length, the diameter of the tumor will determine the number of needles needed along with their exact configuration. However, as a general rule, we found the optimum distance for cryo-needle placement to be between 1.5 and 2 cm. Although our data appear comparable with live porcine experiments, as our data are not in human tissue, it would be reasonable to use a more conservative distance of 1-1.5 cm, which would balance ice ball volume and cell kill temperatures. As mentioned previously, manipulation of duty cycle settings can allow further changes in ice ball size, but with low duty cycle settings, the risk of incomplete coverage of the tumor by the −40°C isotherm increases and thus in routine practice we feel that a setting of 70%-100% is most appropriate.

Intra-operative imaging is also an important factor to consider. In renal cancer, percutaneous surgery allows for direct visualization under computed tomography and magnetic resonance (MR).^22^ The growing low-attenuation lesion has been shown to closely resemble the leading edge (0°C) of the ice ball, whereas MR images provide a superior contrast between the ice ball and the surrounding tissue.^23^ Although in-bore MR techniques exist for the prostate, they are not practical, and thus US is our chosen modality for intra-operative imaging. However, as the US probe is located transrectally, once the ice ball starts to develop, the anterior aspect of the ice ball is no longer visible because of the acoustic shadow cast by its leading edge. It must also be noted that the lethal temperature isotherm may be up to 1 cm beneath this leading edge. As neither computed tomography nor US can predict an ice ball isotherm, MR has a significant advantage in this area.

**Figure 4:**
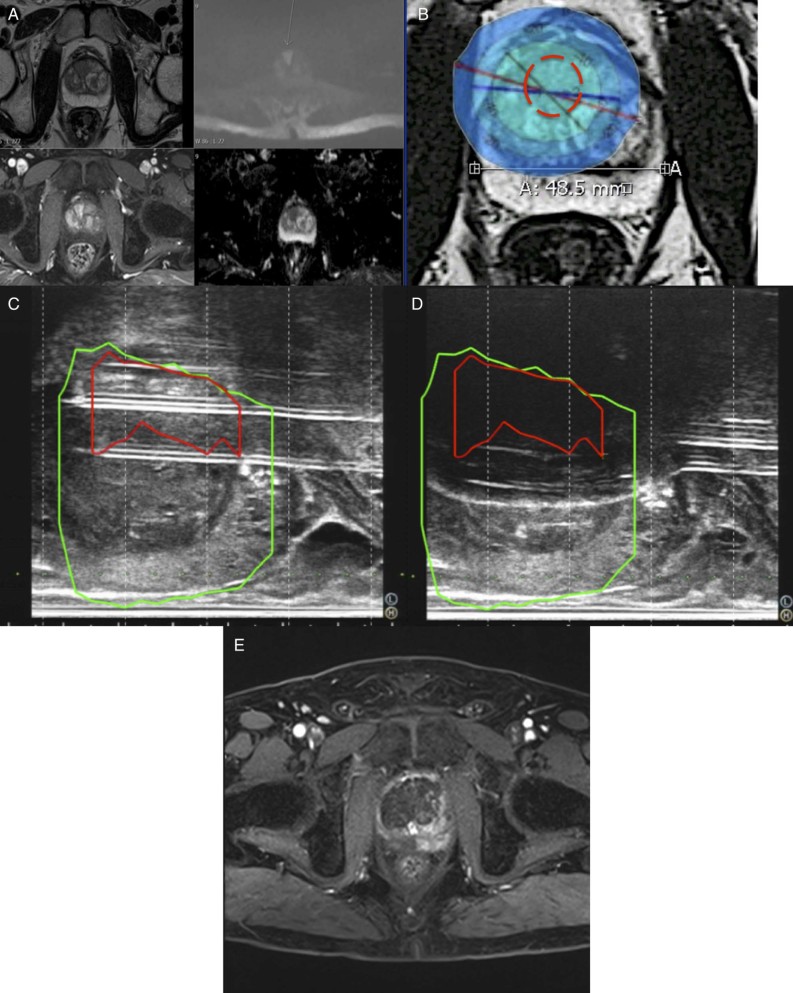
**(A)** Multi-parametric magnetic resonance imaging showing 18 mm max. diameter anterior prostate cancer. **(B)** Overlaid ice ball data, 1 cm apart at a duty cycle of 100% showing adequate coverage by −40°C isotherm over lesion. **(C)** Intra-operative needle placement using Biojet ultrasound (US) fusion system. **(D)** Intra-operative ice ball expansion. **(E)** Dynamic contrast enhanced magnetic resonance (MR) images showing area of necrosis and concordance with preoperative planning. (Color version available online.)

Use of data such as these can help rationalize the number of needles needed to allow adequate coverage of a tumor in either focal or whole-gland prostate or renal cryoablation. With the development of US fusion systems for prostate cancer, further preoperative optimization can be performed by preselecting the needle positions. We have highlighted a case of prostate cancer where use of our data has allowed accurate preoperative planning of needle number, configuration, and duty cycle settings, along with intra-operative use of the Biojet US fusion system^24^ (Fig. 4).

There are, however, some potential limitations in our study that must be highlighted. First, a gel model at 37°C is not human tissue, and the thermal properties may vary. Second, we have not accounted for variations in vascular anatomy or tested configurations around the urethral warmer during prostatic procedures. However, our data might allow significant pre- and intra-operative decisions to be made in the delivery of cryotherapy for ablation of soft-tissue tumors and is the only study of its kind assessing such a large combination of factors. Designing an experimental model to accurately assess all possible combinations of the variables is a challenge. The ideal study would allow real-time monitoring of the ice ball in patients undergoing treatment followed by histologic assessment of the treated lesion. However, because of the number of combinations and ethical considerations needed, this scenario is very difficult if not impossible to create. Third, we also did not assess temperature drop in relation to duration of freeze cycle and used the currently accepted protocol of two 10-minute freeze cycles separated by a 5-minute passive thaw.

We have tried to build an accurate gel model based on the literature, and it appears that our results do compare well with other in vivo studies. Future work will need to focus on modeling ice balls around structures that may lead to a heat sink effect, such as blood vessels and urethral warming catheters. Additionally, there is growing evidence that cryotherapy can lead to both immune stimulatory and inhibitory effects. There may be surgical factors that influence this phenomenon, and Urano et al showed that treatment of single rather than multiple lesions within the liver resulted in the largest immune response.^25^ Additionally, a study on a rat breast carcinoma cell line suggested that the immune response decreased as a larger bulk of tissue was frozen.^26^ Development of animal models in the future, which allow in vivo testing of numerous ice ball configurations, may allow us to gain a further insight into this phenomenon.

## Conclusion

We set out to answer 4 questions: (1) What type of cryo-needle should be adopted? (2) How many needles should be used? (3) Which is the best spatial needle configuration? and (4) Which is the correct duty cycle percentage?

To answer questions 1-3, the length, diameter, and shape of the tumor will ultimately determine the number of needles needed along with their exact configuration. Our results show that the −40°C lethal isotherm is approximately 1 cm inside the leading edge of the ice ball. The optimum distance between cryo-needles was 1.5-2 cm, at duty cycle settings of 70%-100%. At distances further apart or with lower duty cycle settings, ice balls had either a central core >−40°C or an hourglass shape. However, considering that our results are not within live tissue, we feel that a more conservative distance for cryo-needle placement between 1 and 1.5 cm should be adopted for clinical practice.

To answer question 4, although the use of low duty cycle settings is possible, it runs the risk of incomplete coverage of the tumor by the −40°C isotherm, and thus in routine practice we feel that a setting of 70%-100% is most appropriate.

We have presented a large amount of experimental data on ice ball isotherms, which can aid a surgeon in operative planning with regard to needle placement and duty cycle settings to ensure that the lethal isotherm of −40°C adequately covers the tumor.

## Appendix Supplementary Data

Table S1 IceSphere, IceRod, and IceEdge ice ball and isotherm dimensions with varying needle configurations at a duty cycle setting of 100%.
