# Supplementary material for: Modeling Cryotherapy Ice Ball Dimensions and Isotherms in a Novel Gel-based Model to Determine Optimal Cryo-needle Configurations and Settings for Potential Use in Clinical Practice
Source: Urology. 2016 May;91:234–40. doi: 10.1016/j.urology.2016.02.012 (PMC4850244; doi:10.1016/j.urology.2016.02.012)
Supplement: Table S1 — IceSphere, IceRod, and IceEdge ice ball and isotherm dimensions with varying needle configurations at a duty cycle setting of 100%. [file mmc1.docx]

|  |  |  |  |  |  |  |  |  |
| --- | --- | --- | --- | --- | --- | --- | --- | --- |
| IceSphere | # of Needles | Spacing | Area | | | Max Diameter | | |
|  |  |  | 0°C | -20°C | -40°C | 0°C | -20°C | -40°C |
|  |  | [cm] | [cm2] | [cm2] | [cm2] | [cm] | [cm] | [cm] |
|  | 1 | NA | 8.1 | 4.3 | 1.8 | 3.4 | 2.5 | 1.8 |
|  |  |  |  |  |  |  |  |  |
|  | 2 | 1 | 14.6 | 9 | 5.1 | 4.5 | 3.6 | 2.7 |
|  |  | 1.5 | 15.8 | 10 | 5.9 | 4.9 | 4 | 3 |
|  |  | 2 | 16.6 | 10.6 | 6.2 | 5.2 | 4.4 | 3.4 |
|  |  |  |  |  |  |  |  |  |
|  | 3 | 1 | 19 | 12.4 | 7.6 | 5 | 4.1 | 3.3 |
|  |  | 1.5 | 21.1 | 14.2 | 8.9 | 5.5 | 4.6 | 3.6 |
|  |  | 2 | 23.9 | 16.5 | 10.5 | 5.8 | 4.8 | 4.1 |
|  |  |  |  |  |  |  |  |  |
|  | 4 | 1 | 22.7 | 15.4 | 9.6 | 5.5 | 4.5 | 3.6 |
|  |  | 1.5 | 26.4 | 18.3 | 12 | 5.9 | 4.9 | 4.2 |
|  |  | 2 | 29.7 | 21.6 | 14.8 | 6.4 | 5.5 | 4.7 |
|  |  |  |  |  |  |  |  |  |
|  |  |  |  |  |  |  |  |  |
| IceRod | # of Needles | Spacing | Area | | | Max Diameter | | |
|  |  |  | 0°C | -20°C | -40°C | 0°C | -20°C | -40°C |
|  |  | [cm] | [cm2] | [cm2] | [cm2] | [cm] | [cm] | [cm] |
|  | 1 | NA | 9.4 | 4.9 | 2 | 3.6 | 2.6 | 1.8 |
|  |  |  |  |  |  |  |  |  |
|  | 2 | 1 | 17.9 | 11.1 | 6.3 | 5 | 3.9 | 2.9 |
|  |  | 1.5 | 19.2 | 12.3 | 7.3 | 5.4 | 4.3 | 3.4 |
|  |  | 2 | 20 | 13 | 7.7 | 5.7 | 4.7 | 3.7 |
|  |  | 2.5 | 21.2 | 13.9 | 8.1 | 6.2 | 5.2 | 4.2 |
|  |  |  |  |  |  |  |  |  |
|  | 3 | 1 | 22.7 | 15 | 8.8 | 5.5 | 4.4 | 3.5 |
|  |  | 1.5 | 26.1 | 17.8 | 11.2 | 6.1 | 5.1 | 4 |
|  |  | 2 | 27.9 | 19.7 | 13 | 6.2 | 5.2 | 4.4 |
|  |  |  |  |  |  |  |  |  |
|  | 4 | 1 | 26.4 | 17.9 | 11.1 | 5.9 | 4.9 | 3.8 |
|  |  | 1.5 | 30.4 | 21.6 | 14 | 6.3 | 5.3 | 4.4 |
|  |  | 2 | 35 | 26 | 18.1 | 6.9 | 5.9 | 5 |
|  |  |  |  |  |  |  |  |  |
|  |  |  |  |  |  |  |  |  |
| IceEdge | # of Needles | Spacing | Area | | | Max Diameter | | |
|  |  |  | 0°C | -20°C | -40°C | 0°C | -20°C | -40°C |
|  |  | [cm] | [cm2] | [cm2] | [cm2] | [cm2] | [cm2] | [cm2] |
|  | 1 | NA | 13.9 | 8.1 | 4.1 | 4.3 | 3.3 | 2.4 |
|  |  |  |  |  |  |  |  |  |
|  | 2 | 1 | 21.6 | 13.8 | 8 | 5.4 | 4.4 | 3.4 |
|  |  | 1.5 | 23.8 | 15.7 | 9.5 | 5.8 | 4.9 | 3.8 |
|  |  | 2 | 24.9 | 16.3 | 9.9 | 6.2 | 5.2 | 4.2 |
|  |  | 2.5 | 26.1 | 17.6 | 11 | 6.7 | 5.7 | 4.7 |
|  |  |  |  |  |  |  |  |  |
|  | 3 | 1 | 26.6 | 17.7 | 10.5 | 6 | 4.8 | 3.8 |
|  |  | 1.5 | 27.4 | 18.2 | 10.9 | 6.2 | 5.1 | 3.9 |
|  |  | 2 | 35.2 | 25.4 | 17.1 | 6.9 | 5.9 | 4.9 |
|  |  | 2.5 | 32.7 | 22.2 | 13.8 | 6.9 | 5.7 | 4.6 |
|  |  |  |  |  |  |  |  |  |
|  | 4 | 1 | 32.3 | 22.9 | 14.9 | 6.6 | 5.5 | 4.4 |
|  |  | 1.5 | 37.1 | 27.2 | 18.6 | 6.9 | 5.9 | 5 |
|  |  | 2 | 42.4 | 32 | 22.9 | 7.5 | 6.6 | 5.6 |
|  |  | 2.5 | 47.1 | 36.2 | 26.5 | 8.4 | 7.4 | 6.6 |

**Supplementary Table 1.** IceSphere, IceRod, IceEdge ice-ball and isotherm dimensions with varying needle configurations at a duty cycle setting of 100%.
